# Consequences of the Covid-19 virus on individuals receiving homecare services in Norway. A qualitative study of nursing students’ reflective notes

**DOI:** 10.1186/s12912-021-00732-x

**Published:** 2021-10-25

**Authors:** Leslie S. P. Eide, Tove Giske, Britt Moene Kuven, Linda Johansson

**Affiliations:** 1 Department of Health and Social Sciences, Western Norway University of Applied Sciences, Post Box 7030, Inndalsveien 28, 5020 Bergen, Norway; 2 Faculty of Health Studies, VID Specialized University, Bergen. Ulriksdal 10, 5009 Bergen, Norway; 3 Jönköping University, School of Health and Welfare, Institute of Gerontology, Aging Research Network-Jönköping, SE-551 11 Jönköping, Sweden

**Keywords:** Home healthcare, Person-centered care, Nursing education, Reflective notes, Coronavirus

## Abstract

**Background:**

Reflective notes in nursing education can facilitate students’ understanding of how individuals in need of home healthcare services experience unfamiliar situations, such as a pandemic. The aim of this study is to describe the consequences of the COVID-19 virus for individuals receiving homecare services through the eyes of nursing students.

**Methods:**

This is a qualitative descriptive study using content analysis to examine reflection notes from 17 nursing students in their last year of academic studies while undertaking home healthcare service training.

**Results:**

Our study shows students’ reflections on the consequences of the COVID-19 virus on individuals needing home healthcare services and their families. The analysis reveals three categories that described the effect of the virus according to students’ reflections: i) how social life became restricted and only includes the closest family members and home healthcare staff (declining social circle), ii) how family members take on more responsibility to care for the individual and the pronounced impact of this on the day-to-day lives of the individual’s next of kin (expanding responsibility of care), and iii) actions and reactions related to preventing the spread of the virus (dealing with the invisible threat).

**Conclusions:**

Students’ reflection notes show that COVID-19 had major consequences, not only on the individuals receiving home healthcare services, but also on their relatives and on home healthcare staff.

## Background

After COVID-19 was declared a global epidemic [1], Norway implemented its strictest social restrictions since World Word II [2]. COVID-19 is particularly dangerous for frail patients with multiple comorbidities [3]. Once acquired, the virus is associated with increased care needs, hospitalization, and mortality for both home-dwelling and hospitalized patients [4–6]. In Norway, municipalities are responsible for providing healthcare for their inhabitants so they can live at home as long as possible. Home healthcare services include nursing, physiotherapy, occupational therapy, mental healthcare, habilitation, and rehabilitation [7]. Except for physiotherapy, which requires a user fee, healthcare services in Norway are free of charge [7]. Most people receiving healthcare at home are old, frail, and have multiple comorbidities.

Covid-19 was detected in Norway for the first time on 26th of February 2020, and after ten days 169 cases were registered [8] In the first days and weeks after the pandemic was declared, there was uncertainty regarding how to carry out necessary home healthcare nursing in ways that were safe. Personal protective equipment and test capacity were limited in the municipalities [9], causing insecurity for nurses, the individuals receiving homecare and their families. The measures to limit the spread of the virus in March 2020 included a lockdown in Norway that also affected universities, resulting in significant challenges for the clinical training that nursing students were receiving. By the time the restrictions were implemented, third-year nursing students on our campus were completing their last clinical training before graduation. These students had the academic foundation to comprehend some of the consequences that the pandemic could have for those they cared for during their training in home healthcare services. However, they had no particular training in how handle such a critical situation.

Nursing places the person, more than the diagnosis, at the center of the practice [10]. In order to provide meaningful, person-centered care, nurses need an integrated understanding of not only how the body works in health and illness but also what it means for a person to live in a healthy and/or sick body [11]. “Embodiment” means to understand the experience of living in and through the body, that is, to view a person as “embodied” rather than as a person who “has a body” [11]. In this context, it is important to acknowledge the skills and knowledge nurses acquire by using their own bodies. Thus, to execute person-centered care, nurses need to have situated awareness and use their senses to collect information. This can be done through touch, eye contact, and observation while assisting the individual, for instance, with activities of daily living or while assessing the effect of a medicine. Educators can facilitate student learning of person-centered care through reflection, personal knowing, and critical thinking [12, 13]. In the context of home healthcare training, nursing students have the opportunity to exercise person-centered care while meeting individuals in their own environment, likely along with their closest relatives.

Nursing education plays an important role in fostering the attitudes and analytical skills necessary to develop, support, and facilitate person-centered care [13, 14], an approach that is receiving increasing support in primary and specialized healthcare in some Nordic countries [15, 16]. The use of reflective notes in nursing education can facilitate students’ understanding of the living experience of the individuals they encounter. These notes can aid students to gain a deeper understanding of how these individuals navigate the unfamiliar environment of the COVID-19 pandemic and their needs and struggles as they face an unknown threat. Therefore, the aim of this study is to describe the consequences of COVID-19 on individuals receiving homecare services through the eyes of nursing students.

## Methods

### Design

This is a qualitative descriptive study that uses content analysis [17] to examine reflective notes from a group of nursing students in their last year of academic studies while undertaking homecare service training.

### Participants and setting

The study was conducted at a large university located in the western part of Norway with students in four campuses (Bergen, Førde, Stord, and Haugesund). In Norway, full-time undergraduate nursing students complete their professional nursing training in three years (full time), and after acquiring their degree, they are qualified to work as registered nurses.

Participants were third-year nursing students at one of the campuses (Bergen) undergoing their final clinical training in homecare services. In total, 55 students were eligible to participate, and 17 agreed to share their reflective notes with the researchers. Since participation was voluntary and anonymous, demographic data of the students who agreed to take part in the study are not available.

Nursing students provided home healthcare services to a variety of individuals. Most individuals receiving home healthcare services lived in an urban area, but some also lived in more rural communities. Because the students had to anonymize data in their reflective notes, we have limited data regarding the individuals’ age, gender, and diagnosis. The students reported caring for men and women and a couple, and the available information indicates that the patients were 60 years and older.

### Data collection

Due to the measures to limit the spread of the COVID-19 virus, clinical placements for all third-year students on our campus were suspended approximately four weeks after they had started. Once the placement was cancelled, the students were given a compulsory curricular assignment consisting of writing a reflective log (maximum 1200 words). They were instructed to select and describe a situation from their training as the pandemic unfolded. Specifically, they were instructed to reflect on the following: 1) What quarantine at home, isolation at home, and the development of the pandemic in Norway could mean for an individual to whom they provided home healthcare services and the individual’s family; 2) What thoughts, feelings, and reactions the experience of the individual/family in the pandemic situation awaken in you; and 3) How can you as a nursing student help and support this individual/family to have the best possible experience in the pandemic situation? All 55 students received written information about the study and were invited to participate after their reflective logs were graded. Participation was voluntary, and those who wanted to participate sent a written consent form and a copy of their reflective note to one of the members of the research team (BMK). The student who authored the reflection note was made anonymous to other members of the research group.

### Data analysis

To analyze the students’ reflective notes, an inductive, qualitative content analysis was performed following Lindgren, Lundman, and Graneheim [17]. All authors read the reflective notes individually and de-contextualized the text before comparing and discussing findings using an interpretative approach. The process continued with re-contextualization, where reflective notes from all nursing students were abstracted and patterns were identified. One researcher (LJ) had the main responsibility for performing the analysis, but during the process the findings were continuously checked and discussed with the other authors. We sought to identify themes that might emerge from students’ experiences of how the COVID-19 pandemic impacted individuals receiving homecare services. Texts from the reflective notes aligned with the aim of the study were highlighted and moved into a spreadsheet. This step revealed that some of the students had not really reflected on how the virus affected the patients they met during their clinical training; rather, their reflections included a more general perspective of how the virus influenced people. These reflective notes were therefore excluded (*n* = 3), and the analysis continued with the remaining 14. Once meaning units in the text were identified and moved into the spreadsheet, they were given a label and coded according to their content. Similar codes were then sorted together by abstraction, and subcategories were created. Through abstraction, categories were identified by sorting related subcategories together. An example of the analysis process is presented in Table 1. Finally, the findings were presented and discussed by the authors until a consensus was reached.

**Table 1:** Example of the analysis process

Meaning units	Condensed meaning unit	Codes	Subcategory	Category
... they must also step in and be a resource that they would otherwise have received from the municipality. (R9)	The family needs to be the home healthcare resource that the municipality would have provided otherwise.	Increased responsibility of the family	Receiving less support	Expanding the responsibility of care
We meet a daughter who is exhausted, who encounters an everyday life in need of great effort. We meet a daughter who is in an almost impossible dilemma between taking care of herself or taking care of her mother. We meet ourselves in home nursing with a situation that requires more resources and more frequent follow-up, in a situation where a pandemic means that you face a shortage of resources and have fewer people at work to follow up. (R17)	Meets an exhausted daughter and must choose between taking care of herself or of her mother. At the same time, home healthcare is in a difficult situation, with lower available resources and fewer staff due to COVID-19.	Less resources but increased care needs		
By the end of the training period, staff dropouts were a fact, which can lead to the task being reduced, that they do not have as much time for each individual patient, and that the nurses only come to ensure the basic physical need, rather than the psychological need which is the one that increases right now. (R10)	Fewer staff leads to less time with a patient, and only basic physical needs are met.	Focus on essential care	Alteration in care structure	

### Ethical considerations

Ethical approval for the study was obtained from the Norwegian Data Protection Office (NSD 298836). Students’ participation was voluntary, and data from the reflective notes were made anonymous.

## Results

According to the students’ reflective notes, the consequences of the COVID-19 pandemic on individuals receiving homecare services not only affected them but also, to a large extent, their closest family. The individuals in this study received home healthcare services from one to six times, every 24 h, and required help with activities of daily living, such as bathing, dressing, going to the toilet, simple food preparation, and administration of medicines. Procedures such as tapping of pleura liquid, care for venous port for palliative patients, handling and care of tracheostomy, colostomy, and help with assisted feeding through percutaneous endoscopic gastrostomy (PEG) tube were reported by the students. Some individuals were in a palliative phase of cancer, and some had experienced stroke, dementia, mental health issues, or had chronic obstructive pulmonary disease. The individuals needed encouraging conversations, trust building and support to deal with stress and anxiety, and a caring relationship.

**Fig. 1:**
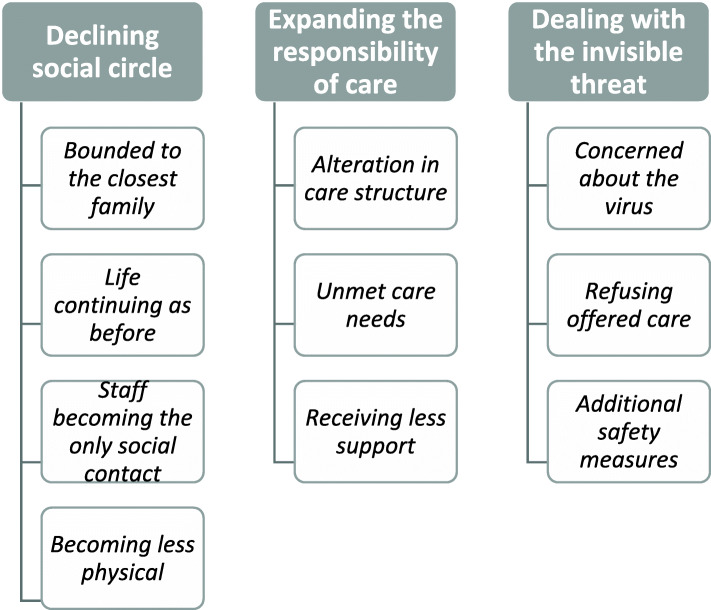
Categories and belonging subcategories

Our analysis revealed three categories that described the effect of the virus according to students’ reflections: i) how social life became restricted and only includes the closest family members and home healthcare staff (declining social circle), ii) how family members take on more responsibility to care for the individual and the pronounced impact of this on the day-to-day lives of the individual’s next of kin (expanding responsibility of care), and iii) actions and reactions related to preventing the spread of the virus (dealing with the invisible threat). The three categories and belonging subcategories (in italics) are presented in more detail below and in Fig. 1.

### Declining social circle

This category contains four subcategories and focuses on how social life became restricted and how it only includes the closest family members and home healthcare staff.

During the pandemic, individuals’ social networks decrease in size and they become *bound to their closest family members*. The individuals meet family members living outside their household and friends less frequently, causing them to feel lonely and cut off from important relationships. This is also true for family members living in the same household, as they must also stop seeing others outside the closest family circle, such as grandchildren. The restrictions that were in place by the time the students wrote their reflective notes required people to stay at home and to limit social contact.

*This is a lady who usually is social and "on the go." She often has visits from family, and she herself goes to visit neighbors and friends. The [individual] told me that she thought it was awkward that she could not get out and around these times.* (R14)

At the same time, family members living outside the household and friends opted to not visit the individual to reduce the chances of infecting him or her. Still, the students indicated that, for some individuals, *life continued as before* because their social arena had already been limited prior to the restrictions due to their illness. However, there were also individuals who had the opportunity and chose to keep performing activities they deemed important and maintained their social life regardless of the pandemic.

*She [the individual] had been really looking forward to this trip and would not let the Corona situation stop her. Even though we told her that she was at risk, she and her family traveled. But travel was not discouraged [by the government] at that time.* (R4)

As interaction with their regular social network declined, sometimes home healthcare *staff became the only social contact* an individual had.

*For [this person] and many others in her situation, the daily contact with the home healthcare services often are the highlight of the day, and the only direct contact they have with other human beings (R10)*

Yet, this social interaction did not provide them with the same gratification as, for instance, meeting their grandchildren. At the same time that social network interactions decreased, physical distancing increased. To reduce the spread of the virus, people avoided touching hands and/or hugging one another, that is, they *became less physical.* This also applied to home healthcare staff, who had to maintain physical distance from the individual’s relatives, as one student explained:

*I experienced it as very painful in this situation that I could not give [the individual’s] wife a hug or to hold her hand, I had to always make sure that I had a good distance to her to be safe*. (R 12)

### Expanding the responsibility of care

This category shows how family members take on more responsibility in terms of caring for the individual receiving home services, and the pronounced impact that these responsibilities have on the day-to-day lives of the individual’s next of kin.

As a result of the pandemic, there was a rapid *alteration in the care structure* for individuals receiving home services. In some cases, only the most needed home healthcare assistance was given because staff members were on sick leave or in quarantine. This affected the dynamic between the individuals and family members sharing the same household because caring for the individual’s health interfered with the relatives’ everyday lives. Some family members could feel home bounded to an environment that resembles a hospital setting, as shown in the following example:

*[they are] walking in a home that has been converted into a hospital, with nurses from the home healthcare services coming and going. (R1)*

Healthcare equipment was more visible in the home, and outdoor support was no longer available because daycare facilities for persons with dementia were closed. The individual’s home began to resemble a healthcare facility that was attended by the closest relatives. One student observed a sudden change in a healthcare plan when someone could not be discharged from the hospital to a short-term care facility, as previously arranged, but was instead discharged directly to the household:

*Due to the situation with COVID-19, immediate action was taken by two doctors who decided that [he] should be discharged [from the hospital] immediately, because the risk [of contracting the virus] was greater at the institution than it was at home. Anyway, [he] wanted to be at home on permanent basis. If [he] contracted the virus, he would probably not survive. It was challenging that [the discharge] took place almost three weeks before it was planned. Therefore, initially a nurse from the short-term facility came with the homecare nurse. The patient and the spouse had an extra person into their home and the homecare nurses/nurse assistant felt insecure about the job they had to do.* (R7)

Regarding changes in healthcare plans, there were also reflective notes showing individuals’ and families’ concerns about *unmet care needs.* Both worried that it would not be possible to receive healthcare when needed. Individuals were anxious about not knowing whether family members could visit them if they had to move to a healthcare facility, while families were concerned about individuals not getting help if needed. A student observed and reflected on how this uncertainty could create a complex situation and an ethical dilemma. In this case, the decreased health condition of the individual resulted in families being forced to make quick decisions about healthcare without really knowing how it would affect them later.

*What will happen if he accepts the place at the nursing home tomorrow? Does this mean that today is the last day he gets to see his children? And his grandchildren? What are the possibilities of them visiting him at the nursing home if his health condition becomes critical? These questions and thoughts came to him and to his daughters when we visited.* (R16)

Further, the pandemic resulted in family members *receiving less support* to manage the individual’s healthcare needs. Before the social restrictions were implemented, family members could meet their own friends. Now, they have to stay at home, not only because of the ongoing restrictions but also because the daycare facilities the individual used to attend are closed and he or she requires constant health supervision at home.

*She [the spouse] needs some time to herself while he is on a short-term facility. It is then she can go shopping, visit friends and travel to warmer places. It is not like she can do these things [now] during the pandemic, but whenever he is at home, she cannot leave his side because he could have a cough attack because of his difficulties swallowing.* (R3)

Despite family members’ understanding of the unexpected changes in healthcare support, these alterations generated feelings of frustration, helplessness, social isolation, and exhaustion. In some cases, the restrictions resulted in individuals becoming more dependent, as they could no longer go out or were forced to rely on others to, for example, manage their medications. This in turn increased the family members’ existing burden. Despite needing even more support to cope with the increased demands of the individual’s healthcare needs, relatives received less help because the resources and number of available healthcare staff were limited.

### Dealing with the invisible threat

This category includes three subcategories describing the actions and reactions related to preventing the spread of the virus.

Some individuals were *concerned about the virus*, and the uncertainty of not knowing if they had contracted it created stress and anxiety, especially if they had other comorbidities:

*[She] is old and has various diagnoses that makes her fall into the risk group. That can certainly be scary enough to think about.* (R15)

The fear was not only related to the individual’s own safety but also to family and friends interacting in the society, who might thus have a higher risk of getting infected. Hence, the individuals were concerned about their family members being carriers of the virus and of potentially infecting them. Watching media reports increased anxiety levels even more. The individuals depended on receiving home healthcare services, and, in some cases, there was an increase in the number of home healthcare staff members they had to be in contact with because of changes in care plans schedules. These changes led to the individuals meeting new home healthcare professionals on a regular basis, creating new and multiple meeting points and increasing their anxiety levels accordingly. In the reflective notes, it was also mentioned that on some occasions the new staff was unsure about how to provide the healthcare needed by some of the individuals.

*… [she] was worried about whether the new [staff] knew the procedures. Several of the nurses had expressed that they had not done these kinds of procedures for a long time. (R1)*

The students reflected about some individuals or their families *refusing offered care,* and for these students it meant that they were not welcomed into the individual’s home*.* This issue arose because members of the household wanted to decrease the number of persons they met and thus reduce the risk of infection. Another reason to *refuse offered care* was that individuals and relatives were aware of the home healthcare staff seeing other patients and the increased risk of infection:

*… she has said she is happy I came to her alone. She is afraid of the risk of infection in connection with the healthcare nurse visiting many others, and that this increases the risk of infection for her.* (R10)

The uncertainty of COVID-19 required *additional safety measures*. Some individuals and their family members controlled the staff’s hygiene routines and how they used personal protective equipment. Students also reflected upon how they, as well as other home healthcare staff, had to take extra precautions and how different scenarios had to be handled because of the restrictions. One student described the struggles with performing cardiopulmonary resuscitation on an unknown person because of the risk of contracting the virus. The fact that staff must now use extra personal protective equipment can sometimes lead to anxiety in individuals and their families, as the safety equipment and gloves symbolize the imminent danger. A student reflected upon this in the following way:

*The safety we often carry with us, the "warm hands," is now hidden behind infection control equipment and synthetic gloves...* (R17)

## Discussion

This study describes the consequences of the COVID-19 pandemic for individuals receiving home healthcare services through the eyes of third-year nursing students, based on an analysis of reflective notes during the first wave of the pandemic. We identified three themes from the data that described the effect of the virus: i) declining social circle, ii) expanding healthcare responsibility, and iii) dealing with an invisible threat. The reflective notes offer insight on how students developed an understanding of the serious physical, social, psychological, and spiritual consequences the virus imposed on home-dwelling individuals and their families. Moreover, the reflective notes provided the students with an opportunity to gain a deeper understanding of how to partner with different individuals and their families during the early stage of the pandemic. Our data also show that students had to take the individual’s values and preferences into consideration when providing home healthcare, which is a key attribute of person-centered care [10, 12].

The first theme in this study refers to a decline in the social networks of individuals and their closest family members. Social isolation and loneliness have a detrimental effect on the health and well-being of people in general and on older patients in particular [18]. The students’ reflective notes show that, at this early stage of the pandemic and despite understanding the need for restrictions to prevent the spread of the virus, students were concerned about the loss of social contact between individuals and their closest relatives, other members of the extended family group, and friends. Similar findings have been reported in other population groups [19] and for previous outbreaks [20]. Also, a Dutch study including persons 65 years and older revealed that the participants become more socially and, especially, emotionally lonely during the COVID-19 pandemic [21]. Online and telephone support have been highlighted during the pandemic as one possible way to reach out to persons who otherwise would not receive psychological and emotional support [20, 22, 23]. However, there might be digital inequality and large differences in the ability to use telemedicine and e-health services. The reflective notes show that social distancing due to the restrictions was not perceived as a negative outcome for all individuals. In these cases, the individuals felt that their health conditions had isolated them from social interaction before the restrictions came into place. Hence, the restrictions had few consequences because, even before the pandemic, they had little contact with others. Our results are consistent with those of another study [24] and might reflect a larger problem that is not covered in this article: loneliness.

Most home-dwelling individuals in need of health homecare services are frail and dependent on the care of others. Frailty, a condition characterized by a decline in functioning across several physiological systems, is associated with increased vulnerability to stressors [25]. Frailty has been linked to numerous adverse outcomes, including increased disability, falls, loneliness, hospitalization and nursing home admission [26]. In Norway, the restrictions to prevent the spread of COVID-19 resulted in unexpected changes in healthcare plans and reduced home healthcare availability. These changes placed an extra burden on individuals and families since only the most crucial homecare assistance was performed, as observed in the present study. Other studies have shown similar results regarding the increased burden of care on other family members [27] [28]. In the long term, these abrupt changes could lead to a rapid decrease in individuals’ physical and mental function and, consequently, to accelerated nursing-home placement. Despite increased interest in the field of frailty, the translation from research to clinical practice remains difficult [26]. This raises an important challenge for universities and colleges educating nurses, as it is imperative for students to learn about person-centered care when frailty is present.

Students identified feelings of fear among home-dwelling individuals and their family members. Because of concerns about contracting the virus, students were not able to provide necessary help, as access to the individuals’ homes was denied. Anxiety related to COVID-19 have been reported in other studies [24, 29], and similar reactions toward healthcare workers were identified during the Ebola [30] and SARS pandemics in the early 2000s [31]. These findings are somewhat contradictory to those presented in the theme *Expanding healthcare responsibility*, but this discrepancy could be explained by the limited knowledge that society in general had about COVID-19 at the time the reflective notes were written as well as the misleading information circulating on social media. The students also described feelings of anxiety and insecurity when, in critical situations, they could not come close physically to individuals and their relatives, as shown in the reflection note about the staffs’ warm hands being hidden behind infection control equipment. This reinforces the fact that some parts of nurses’ bodies, such as their hands, are important instruments of practice [11]. Providing nursing students with the possibility to actively reflect in either verbal or written form when encountering new and complex situations can be useful in acknowledging their embodied actions and the impact that their actions might have on person-centered care now and later in their professional lives.

Overall, this study revealed that the students observed both physical, social, psychological, and spiritual consequences experienced by individuals and their closest family members as a result of the pandemic. This is in line with person-centered care, where the individual’s biomedical sphere is as important as the psychosocial, spiritual, and relational areas. Reflection allows students to think about their own experiences, values, and beliefs [32], and by doing so students can better understand person-centered care [12]. Reflection as a learning tool offers students the possibility to think through the situation, and what they experience helps to create meaning in what happened. Reflection after action provides a certain distance to the situation, which can lead to a better understanding of what we thought at the time and how we acted in a circumstance [33]. Reflection can be carried out at different levels, from a more superficial account of what happened to a deeper and more critical reflection. Such deeper reflection provides an opportunity to develop personal and professional insights that might lead to transforming professional practice. Reflection is important at three different times during an action [33, 34]. First, we can reflect before we enter a situation and imagine what could happen. Second, when we are in a situation, we can reflect about what is happening as it happens, which helps us to stay actively present in the situation and consciously act as well and wisely as possible. The last form of reflection is reflecting after a situation has finished. It is this last form of reflection that the students in this study were invited to carry out. Through a written log, students revealed their constant efforts to understand the needs of the individual they provided home healthcare to in a period characterized by uncertainty. They also wrote about the narrowing of the individuals’ social circles and of the increased healthcare responsibilities placed on families while dealing with the invisible threat of COVID-19.

There are some limitations in this study that need to be acknowledged. First, the study was conducted at a single campus, and students’ placements were restricted to one city and the neighboring municipalities. Thus, the perceptions of the students may only be applicable to one site. Second, the study was conducted during a period when there was little knowledge about COVID-19. Therefore, the government and the rest of the society were on maximum alert, and the students’ reflections were largely based on own experiences, perceptions, and thoughts rather than on evidence-based knowledge. Another limitation relates to the fact that the management of the COVID-19 pandemic differed in various parts of Norway depending on the levels of infection in a specific area. Further, the management of the virus was different in other countries. Additionally, our sample was based on a convenience sampling of students who wanted to participate in the study. Since the original purpose of the reflection was education instead of research, we were unable to influence the questions to be answered. This resulted in three reflective notes being excluded, as they did not focus on specific situations but rather on how the pandemic might affect people from a broader perspective.

## Conclusions

Building on third-year students’ reflective notes during the early stages of the pandemic, this study revealed several consequences of COVID-19 on individuals receiving home healthcare services in Norway. As social life became more restricted, the social circles of many individuals became smaller. Due to quarantine, the number of healthcare staff became limited, so the criteria for who could receive homecare services became stricter. At the same time, some individuals and their families did not want to receive homecare due to a fear of contracting the virus. In some families, the next of kin had to take on a more substantial role in healthcare as part of dealing with the invisible threat of COVID-19.

The study results indicate that reflective notes provide an important medium for students to address and focus on their clinical experiences. In our study, these notes promoted a better understanding of person-centered care and helped the students create connections between theory and clinical practice.

## Data Availability

The datasets used during this study are available from the corresponding author on reasonable request.
